# CXCR4 antagonism ameliorates leukocyte abnormalities in a preclinical model of WHIM syndrome

**DOI:** 10.3389/fimmu.2024.1468823

**Published:** 2024-11-11

**Authors:** Lilian Roland, Chi Huu Nguyen, Katarina Zmajkovicova, Mélanie Khamyath, Maria Kalogeraki, Bérénice Schell, Vanessa Gourhand, Vincent Rondeau, Zeina Abou Nader, Halenya Monticelli, Barbara Maierhofer, Robert Johnson, Arthur Taveras, Marion Espéli, Karl Balabanian

**Affiliations:** ^1^ Université Paris Cité, Institut de Recherche Saint-Louis, INSERM U1160, Paris, France; ^2^ OPALE Carnot Institute, The Organization for Partnerships in Leukemia, Hôpital Saint-Louis, Paris, France; ^3^ X4 Pharmaceuticals (Austria) GmbH, Vienna, Austria; ^4^ X4 Pharmaceuticals Inc, Boston, MA, United States

**Keywords:** WHIM syndrome, primary immunodeficiency, chronic neutropenia, CXCR4 antagonism, preclinical study, neutrophil function

## Abstract

**Background:**

WHIM (Warts, Hypogammaglobulinemia, Infections, and Myelokathexis) syndrome is an ultra-rare, combined primary immunodeficiency and chronic neutropenic disorder characterized by a range of clinical presentations, including peripheral neutropenia, lymphopenia, and recurrent infections. WHIM syndrome is most often caused by gain-of-function mutations in the gene encoding C-X-C chemokine receptor 4 (CXCR4). As such, inhibition of CXCR4 with XOLREMDI^®^ (mavorixafor), an orally bioavailable CXCR4 antagonist, demonstrated clinically meaningful increases in absolute neutrophil and lymphocyte counts and concomitant reduction in infections in patients with WHIM syndrome, resulting in its recent U.S. Food and Drug Administration approval. The impact of CXCR4 antagonism on other aspects of the pathobiology in WHIM syndrome, such as lymphopoiesis and leukocyte trafficking between primary and secondary lymphoid organs, is less understood.

**Methods:**

In the current study, the effects of CXCR4 antagonism on leukocyte trafficking and distribution in primary and secondary lymphoid organs were investigated in a mouse model of WHIM syndrome carrying the heterozygous *Cxcr4^1013^* mutation. *Cxcr4^+/1013^* and *Cxcr4* wild-type mice received the orally bioavailable CXCR4 antagonist X4-185. Blood, spleen and bone marrow samples were collected for numeration, flow cytometry, and functional studies.

**Results:**

*Cxcr4^+/1013^* mice exhibited profound peripheral blood leukopenia as seen in patients with WHIM syndrome. CXCR4 antagonism corrected circulating leukopenia and mobilized functional neutrophils without disrupting granulopoiesis in the bone marrow of *Cxcr4^+/1013^* mice. Furthermore, *Cxcr4^+/1013^* displayed aberrant splenic T and B-cell counts and frequency. Treatment with X4-185 normalized splenic T-cell abnormalities, correcting the reduced CD8^+^ T-cell numbers, restoring the CD4/CD8 T-cell ratio, and ameliorating peripheral blood T-cell lymphopenia. In addition, CXCR4 antagonism was able to correct the abnormal frequencies and numbers of splenic marginal zone and follicular B cells in *Cxcr4^+/1013^* mice, and ultimately normalize B-cell lymphopenia in the peripheral circulation.

**Conclusions:**

Our study provides comprehensive evidence that oral dosing with a CXCR4 antagonist can effectively correct WHIM-associated neutrophil and lymphocyte abnormalities in a mouse model of WHIM syndrome. These findings extend our understanding of how targeting the dysregulated CXCR4 signaling pathway can ameliorate the pathogenesis of WHIM syndrome.

## 1 Introduction

WHIM syndrome is an ultra-rare, combined, primary immunodeficiency and chronic neutropenic disorder characterized by the presence of any of four key diagnostic features: Warts, Hypogammaglobulinemia, Infections, and Myelokathexis (1–4). Patients may have diverse clinical presentations, including peripheral blood neutropenia and lymphocytopenia, recurrent and sometimes severe bacterial infections, variable hypogammaglobulinemia, increased susceptibility to human papillomavirus (HPV) and Epstein-Barr virus (EBV) infections, and increased risk of malignancy (2, 3, 5, 6). WHIM syndrome is most often caused by gain-of-function (GOF) mutations in the gene encoding C-X-C chemokine receptor 4 (CXCR4) (3, 7–10), a master regulator of immune cell trafficking, homeostasis, and organogenesis (11, 12). GOF mutations cause impaired CXCR4 receptor internalization and desensitization, prolonged downstream signaling, and enhanced chemotaxis in response to its ligand, C-X-C chemokine ligand 12 (2, 3, 13). These altered responses lead to abnormal retention of neutrophils in the bone marrow (BM) and reduce their egress to peripheral blood (2, 10). Moreover, CXCR4 GOF mutations not only affect neutrophils but also B- and T-cell lymphopoiesis and their peripheral trafficking and localization, resulting in B and T-cell lymphopenia (2, 3, 14–18).

As CXCR4 GOF mutations play a central role in the pathogenesis of WHIM syndrome, correction of aberrant CXCR4 signaling using CXCR4 antagonists including plerixafor and mavorixafor has been explored as a potential therapeutic approach for the treatment of WHIM syndrome (19–24). In a placebo-controlled, double-blind Phase 3 study (NCT03995108), mavorixafor demonstrated clinically meaningful increases in absolute neutrophil and lymphocyte counts in patients with WHIM syndrome, resulting in the recent U.S. Food and Drug Administration (FDA) approval of XOLREMDI^®^ (mavorixafor). In addition, treatment with mavorixafor resulted in a reduction of the annualized infection rate in patients with WHIM syndrome (24). Characterizing the pathobiology in tissue compartments of patients with WHIM syndrome (such as B- and T-cell lymphopoiesis and leukocyte trafficking between primary and secondary lymphoid organs) remains a challenge due to the invasiveness of these procedures. Alternatively, preclinical studies in animal models of WHIM syndrome may be used to provide insights into potential WHIM pathogenesis in tissues and the impact of CXCR4 antagonism on these processes.

In previously reported preclinical studies, acute inhibition of CXCR4 was associated with transient increases in neutrophils, and B- and T-cell counts in the peripheral blood of mice harboring the heterozygous *Cxcr4^1013^* mutation (15, 18). However, whether prolonged CXCR4 antagonism could correct leukocyte trafficking and distribution defects in primary and secondary lymphoid organs in this WHIM mouse model remained unclear. In a separate knock-in WHIM mouse model harboring the *Cxcr4^1000^* mutation, chronic CXCR4 inhibition by the orally bioavailable CXCR4 antagonist X4-185 was shown to normalize B cell alteration in BM and B-cell counts in circulation and in secondary lymphoid organs (17). The impact of chronic CXCR4 inhibition on splenic B-cell compartmentalization, as well as granulopoiesis and neutrophil trafficking in this second WHIM mouse model, however, was not described. In addition, whether neutrophil function is impaired and whether CXCR4 antagonist treatment can restore normal neutrophil function in these WHIM mouse models have not been investigated. The current study aimed to comprehensively investigate the effects of chronic dosing with an orally bioavailable CXCR4 antagonist on the WHIM-associated leukocyte dysfunctions in primary and secondary lymphoid organs exhibited by mice carrying the heterozygous *Cxcr4^1013^* mutation.

## 2 Methods

### 2.1 Mouse models

Mice harboring the heterozygous *Cxcr4^+/1013^* mutation were generated as previously described (15, 16, 25). This mouse model was selected because it bears a point mutation in the second exon of *Cxcr4*, which is frequently observed in patients with WHIM syndrome, and was inserted using a knock-in strategy. Littermate wild type (WT) mice were used as controls in all experiments. All mice were 8 -16 weeks old. Daily observation was performed to ensure that no animal was left in a state of pain or suffering during experimentation.

All *in vivo* mouse experiments were conducted at Université Paris Cité, Institut de Recherche Saint-Louis (Paris, France) from March 2022 to May 2023. Studies were conducted in compliance with the European Union guide for the care and use of laboratory animals, which have been reviewed and approved by an institutional review committee (Comité d’éthique Paris-Nord N°121, France).

### 2.2 CXCR4 antagonist treatment

*Cxcr4^+/1013^* and *Cxcr4^WT^* mice received CXCR4 antagonist X4-185 at 10 mg/kg/day (X4 Pharmaceuticals) (17, 26) or vehicle daily for 7 or 28 days in a row (5 days on/2 days off). Vehicle only was given as control in all experiments (H20-NaCl-0.4% pH4 or phosphate-buffered saline [PBS]). X4-185 is an orally bioavailable, small molecule CXCR4 antagonist of molecular weight ~400 g/mol. In internal control experiments, mice were injected intraperitoneally with plerixafor (AMD3100) (5 mg/kg/day) (Sigma-Aldrich, Darmstadt, Germany) for 21 days (15, 25, 27). Mice were bled 7 days before the start of the experiments, 3 hours after the first dose, then every 7 days 3 hours after the dose. Euthanasia was performed by increasing gradient of CO_2_. At sacrifice, BM, spleens, and blood were harvested and processed for numeration on an MS9-5 counter (Melet Schloesing, Laboratoires, Osny, France), and flow cytometry. BM cells were extracted by centrifugation from intact femurs, tibia, and hips to separate marrow and bone fractions. Spleens were manually isolated. Cell collection was performed in PBS supplemented with 2% fetal bovine serum and filtered through a 70-μm nylon strainer to remove debris and fat. The peripheral blood was collected by submandibular puncture. Red blood cell lysis was performed using an ammonium-chloride-potassium buffer.

### 2.3 Flow cytometry

Single cell suspensions were stained with appropriate antibodies in PBS supplemented with 2% bovine serum albumin and 2 mM ethylenediaminetetraacetic acid for cell surface staining. The list of antibodies used in the study is shown in **Supplementary Table S1**. Analyses were carried out on an LSRII Fortessa flow cytometer (BD Biosciences, Franklin Lakes, NJ, USA), and data were analyzed with the FlowJo software (TreeStar, Ashland, OR, USA).

### 2.4 Oxidative burst assay

To determine Reactive Oxygen Species (ROS) levels, whole blood was loaded with 0.5 µg/mL Dihydrorodamine 123 (cat#D1054, Sigma-Aldrich) and incubated for 15 minutes at 37°C. Subsequently, 200 nM phorbol 12-myristate 13-acetate was added, and the samples were incubated for an additional 45 minutes at 37°C to induce the ROS production. For unstimulated samples, incubation with PBS was performed. Then, 2 mL of red blood cell lysis buffer was added, and the mixture was incubated at room temperature. After 10 minutes, the reaction was halted by adding 4 mL of PBS, and samples were centrifuged at 200 g for 5 minutes. The red blood cell lysis procedure was repeated a second time. The fluorescence levels were measured using flow cytometry (BD FACSCanto™ II, BD Biosciences). The gating strategy of granulocytes was based on forward and side scatter plots of the lysed whole blood.

### 2.5 Bioparticle uptake (phagocytosis assay)

To quantify phagocytic activity, pHrodo™ BioParticles™ Conjugates for Phagocytosis (Cat# P35367, Thermo Fisher Scientific, Waltham, MA, USA) was used. Whole blood was incubated with pHrodo BioParticles for 15 minutes at 37°C. Negative controls were incubated for 15 minutes on ice. Then, all the samples were placed on ice to stop the uptake process. Subsequently, 2 mL of red blood cell lysis buffer was added, and the mixture was incubated on ice for 15 minutes. The reaction was halted by adding 4 mL of PBS and samples were centrifuged at 200 g for 5 minutes. The fluorescence levels were measured using flow cytometry (BD FACSCanto™ II). The gating strategy of granulocytes was based on forward and side scatter plots of the lysed whole blood.

### 2.6 Statistical analysis

The data were presented as mean ± standard error of the mean (SEM) and the number of mice per experiments was indicated in the figure legends. All statistical analyses were conducted using Prism software (GraphPad Software, Boston, MA, USA). The significance of differences between two independent groups was calculated using Mann-Whitney test. P values <0.05 were considered statistically significant.

## 3 Results

### 3.1 CXCR4 antagonism corrects peripheral blood leukopenia in *Cxcr4^+/1013^* mice

**Figure 1:**
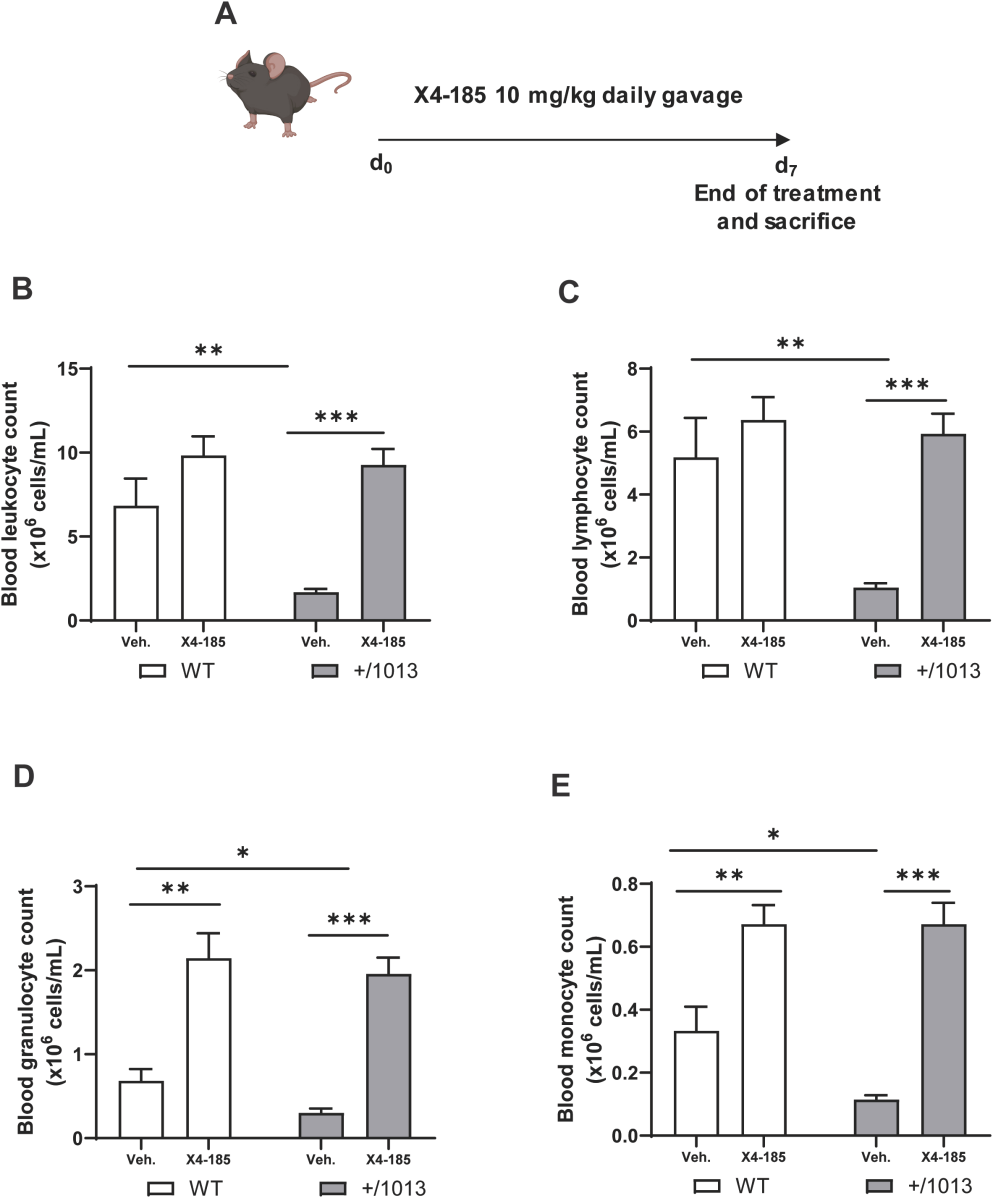
CXCR4 antagonism corrects the peripheral panleukopenia in *Cxcr4^+/1013^* mice. **(A)** Experimental design for CXCR4 signaling inhibition by CXCR4 antagonist X4-185 in WT and *Cxcr4^+/1013^* mice. Absolute **(B)** leucocyte, **(C)** lymphocyte, **(D)** granulocyte, and **(E)** monocyte counts were determined in the blood of WT and *Cxcr4^+/1013^* mice 3 hours after the last dose on day 7. Data (mean + SEM) were from two independent experiments with 6–10 mice per group. Statistics were calculated using the nonparametric Mann-Whitney test, two-sided. *p < 0.05, **p < 0.01, ***p < 0.0001. CXCR4, C-X-C chemokine receptor 4; d, day; SEM, standard error of the mean; Veh., vehicle; WT, wild-type.

It was shown that transient inhibition of CXCR4 signaling reversed circulating leukopenia in *Cxcr4^+/1013^* mice (15). However, whether CXCR4 antagonism durably corrects leukopenia in *Cxcr4^+/1013^* mice following chronic administration has not been investigated. To this end, *Cxcr4^+/1013^* and littermate WT mice were treated with vehicle or the orally bioavailable CXCR4 antagonist X4-185 by daily gavage at 10 mg/kg/day for 7 days, and blood cell counts were measured 3 hours after the last dose (**Figure 1A**). *Cxcr4^+/1013^* mice exhibited profound circulating panleukopenia, with lymphocytes being the most impacted leukocyte subtype. Specifically, *Cxcr4^+/1013^* mice had 75%, 80%, 56% and 66% fewer leukocytes, lymphocytes, granulocytes, and monocytes, respectively, than their WT littermates (**Figures 1B–E**). Membrane expression levels of CXCR4 on neutrophils, B cells, CD4^+^-T cells and CD8^+^-T cells from peripheral blood and BM were similar between *Cxcr4^+/1013^* and WT mice (**Supplementary Figures S1A, B**), in line with our previous reports (15). This finding suggests that peripheral blood leukopenia observed in *Cxcr4* ^+/1013^ mice is likely due to CXCR4 GOF rather than changes in CXCR4 expression. CXCR4 antagonism led to a significant increase in absolute number of all these subsets in *Cxcr4^+/1013^* mice (**Figures 1B–E**; **Table 1**). While CXCR4 antagonism corrected peripheral blood leukopenia in *Cxcr4^+/1013^* mice (**Figures 1B–E**), it did not appear to affect the count of red blood cells or platelets in both *Cxcr4^+/1013^* and WT mice (**Supplementary Figures S2A, B**).

**Table 1:** Effects of CXCR4 antagonist X4-185 on blood cell counts in WT and *Cxcr4^+/1013^* mice.

Cell type	WT mice		Cxcr4^+/1013^ mice	
	Mean fold-increase	p-value	Mean fold-increase	p-value
Leukocyte	1.4	0.1270	5.5	0.0006
Granulocyte	3.1	0.0017	6.5	0.0006
Mature Neutrophil	2.7	0.0012	5.9	0.0002
Monocyte	2.0	0.0093	5.9	0.0006
Lymphocyte	1.2	0.3124	5.7	0.0006
B cells	1.5	0.1807	19.1	0.0006
CD4-T cells	1.2	0.8357	2.4	0.0262
CD8-T cells	2.0	0.5093	7.3	0.0041

Mean fold-increase and p-value were compared with vehicle-treated mice. CXCR4, C-X-C chemokine receptor 4; WT, wild-type.

Altogether, these results indicate that CXCR4 antagonism corrected the peripheral blood leukopenia in *Cxcr4^+/1013^* mice.

### 3.2 CXCR4 antagonism mobilizes functional neutrophils to peripheral blood

**Figure 2:**
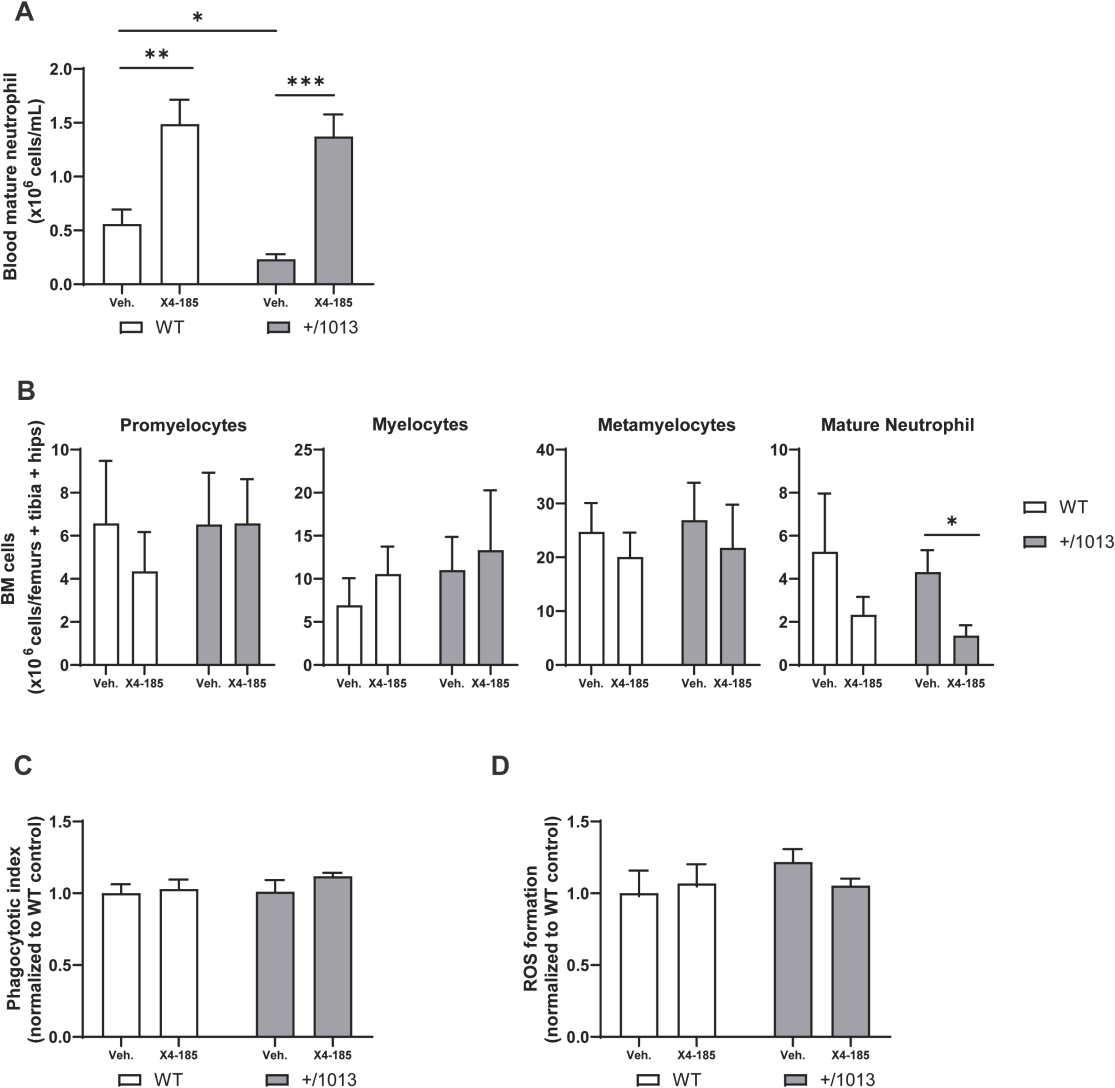
CXCR4 antagonism mobilizes neutrophils in the peripheral blood and preserves their function. **(A)** Absolute counts of mature neutrophils in the blood and **(B)** granulocyte subset cell counts in BM were determined 3 hours after the last dose on day 7. **(C)** Blood neutrophils were harvested 3 hours after the last dose on day 7, incubated with pHrodo BioParticles, and phagocytotic uptake was measured. **(D)** Blood neutrophils were harvested at 3 hours after the last dose on day 7, stimulated with phorbol 12-myristate 13-acetate, and ROS formation was measured. Data (mean + SEM) were from two independent experiments with 6–10 mice per group **(A, B)** and from one experiment with three to four mice per group **(C, D)**. Statistics were calculated using the nonparametric Mann-Whitney test, two-sided. *p < 0.05, **p < 0.01, *** p < 0.001. BM, bone marrow; CXCR4, C-X-C chemokine receptor 4; ROS, reactive oxygen species; SEM, standard error of the mean; Veh., vehicle; WT, wild-type.

Next, we explored the effect of CXCR4 antagonism on granulopoiesis and neutrophil function. *Cxcr4^+/1013^* mice were neutropenic, displaying 61% less circulating mature neutrophils than WT mice (**Figure 2A**). The observed neutropenia in *Cxcr4^+/1013^* mice was not associated with defective granulocyte maturation nor accumulation of mature neutrophils in the BM (**Figure 2B**). Treatment with CXCR4 antagonist significantly increased the number of mature neutrophils in the blood of *Cxcr4^+/1013^* mice (**Figure 2A**; **Table 1**) while, in parallel, decreased the number of mature neutrophils in BM (**Figure 2B**). A similar tendency was observed in WT mice. These findings support BM as an important reservoir for CXCR4 antagonist-mobilized neutrophils, in line with other reports (27, 28). It is worth noting that treatment with CXCR4 antagonist did not affect granulocyte subset cell counts (promyelocytes, myelocytes and metamyelocytes) in BM of both *Cxcr4^+/1013^* and WT mice, indicating that neutrophil differentiation and maturation remained intact during treatment (**Figure 2B**).

At the functional level, blood *Cxcr4^+/1013^* neutrophils exhibited similar capacities to conduct phagocytosis of *S. aureus* BioParticles or to produce ROS compared to their WT counterparts, and these functions were not altered with CXCR4 antagonist treatment (**Figures 2C, D**). These findings support the notion that the enhanced risk of recurrent infections due to pathogenic bacteria in patients with WHIM syndrome is driven by their low leukocyte counts in peripheral blood rather than reduction of phagocytic or ROS neutrophil functions.

Altogether, these results show that CXCR4 antagonism corrected peripheral blood neutropenia and mobilized functional neutrophils, likely from the BM, without impairing BM granulopoiesis in *Cxcr4^+/1013^* mice.

### 3.3 CXCR4 antagonism normalizes the splenic T-cell compartment in *Cxcr4^+/1013^* mice

**Figure 3:**
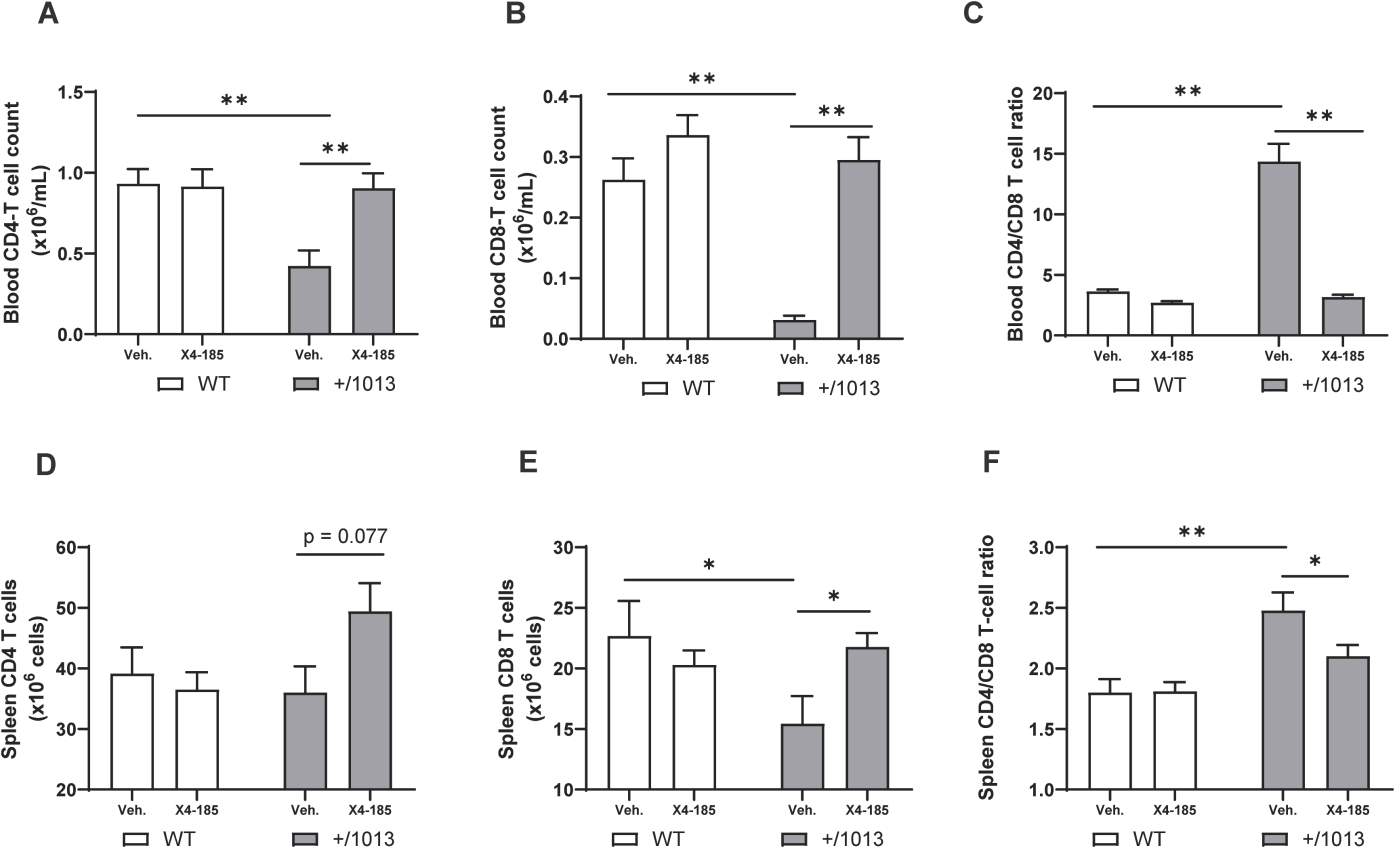
CXCR4 antagonism normalizes the splenic T-cell compartment in *Cxcr4^+/1013^* mice. Absolute **(A)** CD4^+^ T-cell counts, **(B)** CD8^+^ T-cell counts, and **(C)** CD4/CD8 T-cell ratio were determined in the blood 3 hours after the last dose on day 28. Total **(D)** CD4 T-cell numbers, **(E)** CD8 T-cell numbers, and **(F)** CD4/CD8 T-cell ratio were determined in the spleen of WT and *Cxcr4^+/1013^* mice after 28 days of X4-185 treatment. Data (mean + SEM) were from two independent experiments with 6–10 mice per group. Statistics were calculated using the nonparametric Mann-Whitney test, two-sided. *p < 0.05, **p < 0.01. CXCR4, C-X-C chemokine receptor 4; SEM, standard error of the mean; Veh., vehicle; WT, wild-type.

CXCR4 GOF mutations are associated with T-cell lymphopenia in mice, which may result from alteration in T-cell trafficking through secondary lymphoid organs and/or accumulation of T cells in primary lymphoid organs (15–18). It has been shown that transient blockade of CXCR4 signaling resulted in a reversion of circulating T-cell lymphopenia in *Cxcr4^+/1013^* mice (15, 18). However, the impact of CXCR4 antagonism on T cell abnormalities within the secondary lymphoid organs of this WHIM mouse model has not been thoroughly investigated. In the current study, we focused on investigating whether chronic administration of CXCR4 antagonist could modulate T-cell abnormalities in the spleen and peripheral blood of *Cxcr4^+/1013^* mice. In agreement with a recent report (18), *Cxcr4^+/1013^* mice exhibited severe T-cell lymphopenia that affected more CD8^+^ than CD4^+^ T cells, which were reduced by 82% and 55%, respectively, compared with WT mice (**Figures 3A, B**; **Supplementary Figures S3A, B**). Accordingly, circulating CD4/CD8 T-cell ratios were increased by ~3-fold in *Cxcr4^+/1013^* mice compared to WT mice (**Figure 3C**; **Supplementary Figure S3C**). CXCR4 antagonism corrected peripheral blood CD4^+^ and CD8^+^ T-cell lymphopenia in these mice and even restored the circulating CD4/CD8 T-cell ratios to WT levels after 7 days (**Supplementary Figures S3A–C**; **Table 1**) and 28 days of treatment (**Figures 3A–C**).

Next, we quantified CD4^+^ and CD8^+^ T cells in the spleen of WT and *Cxcr4^+/1013^* mice. *Cxcr4^+/1013^* mice had lower CD8^+^ T-cell numbers in the spleen, while CD4^+^ T-cell numbers remained unchanged (**Figures 3D, E**; **Supplementary Figures S3D, E**). Splenic CD4/CD8 T-cell ratios were also increased by ~1.4-fold compared with WT mice (**Figure 3F**; **Supplementary Figure S3F**). Treatment with CXCR4 antagonist for 7 days did not appear to modulate splenic T cell abnormalities (**Supplementary Figures S3D, E**). However, prolonged CXCR4 antagonism for 28 days normalized splenic CD8-T cell numbers in *Cxcr4^+/1013^* mice and restored splenic CD4/CD8 T-cell ratios to WT levels (**Figures 3E, F**). In contrast, this prolonged treatment had no impact on splenic T-cell composition in WT mice (**Figures 3D–F**).

Our data thus indicate that prolonged CXCR4 antagonism can correct splenic T-cell abnormalities and normalize T-cell lymphopenia in peripheral blood in *Cxcr4^+/1013^* mice.

### 3.4 CXCR4 antagonism normalizes the splenic B-cell compartment in *Cxcr4^+/1013^* mice

**Figure 4:**
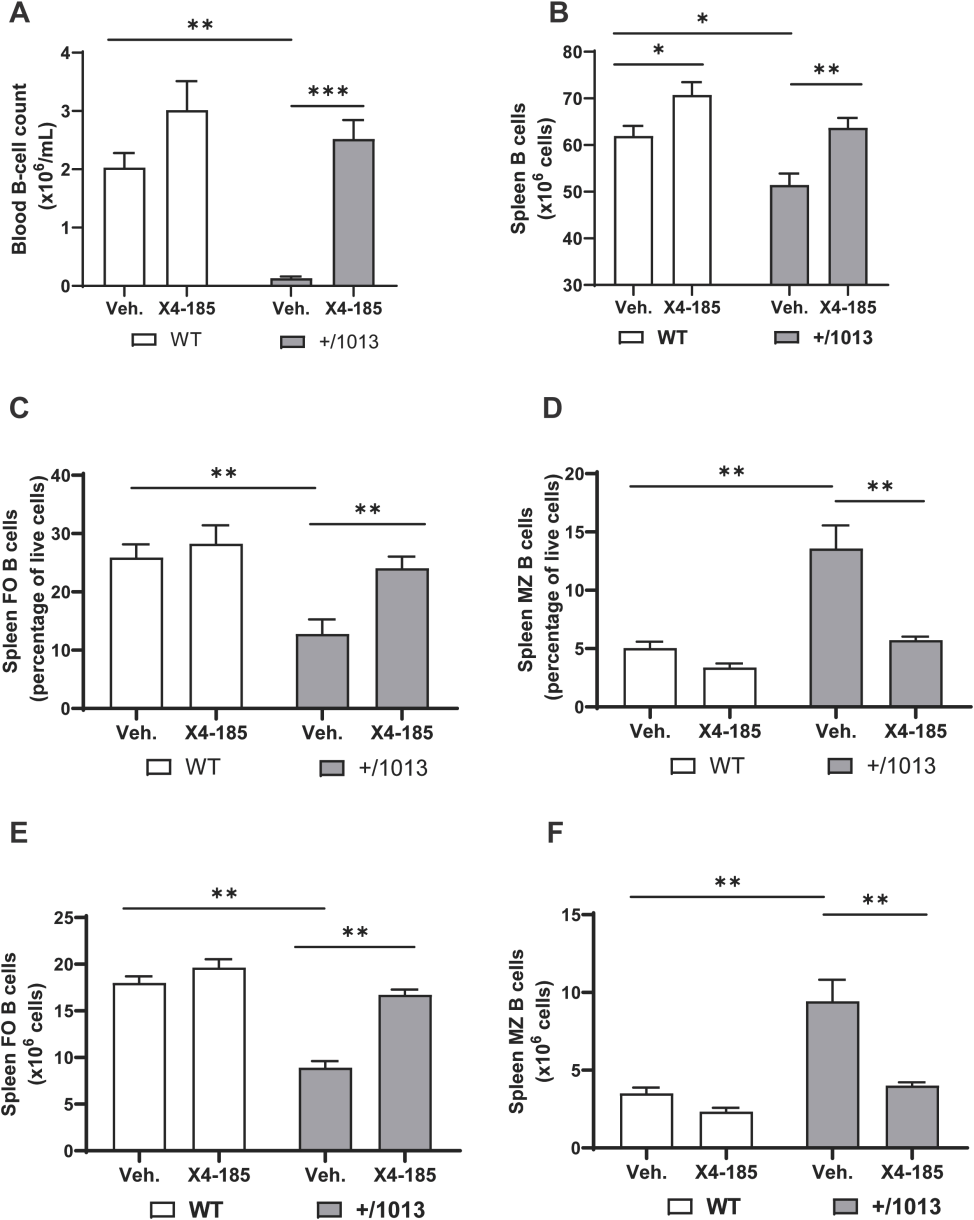
CXCR4 antagonism normalizes the splenic B-cell compartment in *Cxcr4^+/1013^* mice. **(A)** Absolute B-cell counts were determined in the blood of WT and *Cxcr4^+/1013^* mice 3 hours after the last dose on day 7. **(B)** Total splenic B-cell number in WT and *Cxcr4^+/1013^* mice after 28 days of X4-185 treatment. The frequency of **(C)** FO or **(D)** MZ B cells and the total numbers of **(E)** FO or **(F)** MZ B cells were determined in the spleen of WT and *Cxcr4^+/1013^* mice after 28 days of X4-185 treatment. Data (mean + SEM) were from two independent experiments with 6–10 mice per group. Statistics were calculated using the nonparametric Mann-Whitney test, two-sided. *p < 0.05, **p < 0.01, ***p < 0.001. CXCR4, C-X-C chemokine receptor 4; FO, follicular; MZ, marginal zone; SEM, standard error of the mean; Veh., vehicle; WT, wild-type.

CXCR4 GOF mutations alter B-cell development and trafficking resulting in B-cell lymphopenia in mice (15–17). Consistent with these observations, we found that *Cxcr4^+/1013^* mice had severe B-cell lymphopenia in peripheral blood (**Figure 4A**). Treatment with a CXCR4 antagonist for 7 days resulted in an increased B-cell count in *Cxcr4^+/1013^* mice that reached levels seen in WT mice (**Figure 4A**; **Table 1**). Consistent with a previous report (15), IgM levels were slightly higher in *Cxcr4^+/1013^* mice compared to WT mice, while IgG1 levels were comparable to those in WT mice. Chronic treatment with CXCR4 antagonist had no significant impact on IgM and IgG1 levels (**Supplementary Figures S4A, B**).

The observed normalization of circulating B-cell count following CXCR4 antagonist treatment may result from correction of defective B-cell development and/or redistribution of B cells from primary and secondary lymphoid organs. An analysis of spleens from *Cxcr4^+/1013^* mice revealed multiple defects, including decreased spleen weight (~42%), cellularity (39%) and total B cell count (17%) compared with WT mice (**Supplementary Figures S5A, B**; **Figure 4B**) (15). Chronic dosing with CXCR4 antagonist (28-days of treatment) rescued total splenic B -cell number in *Cxcr4^+/1013^* mice to levels observed in WT mice (**Figure 4B**).

It is well established that immature B cells migrating from the BM emerge in the spleen as transitional B cells and further develop into mature marginal zone (MZ) and follicular (FO) B cells (15). We questioned whether this process and distribution of B-cells were defective in *Cxcr4^+/1013^* mice and whether CXCR4 antagonism could correct any related defects observed. As previously reported (15), the frequency and number of FO B cells in *Cxcr4^+/1013^* mice were reduced by >50% compared with WT mice (**Figures 4C, E**). In contrast, the frequency and number of MZ B cells in *Cxcr4^+/1013^* mice were significantly increased compared with WT mice (**Figures 4D, F**). Chronic treatment with the oral CXCR4 antagonist X4-185 for 28 days resulted in correction of both frequency and absolute number of FO and MZ B cells to levels seen in WT mice (**Figures 4C–F**). Reminiscent of previous works (17, 27), no changes in the frequency and counts of splenic FO and MZ B cell were observed in the spleen of WT mice. Interestingly, chronic treatment with plerixafor (AMD3100), an injectable CXCR4 antagonist that has a shorter receptor occupancy time and more limited tissue distribution (as measured by volume of distribution) compared with the orally bioavailable CXCR4 antagonist X4-185 (**Supplementary Table S2**; **Supplementary Figure S6**), was unable to correct splenic FO and MZ B-cell defects observed in *Cxcr4^+/1013^* mice over a 21-day treatment period (**Supplementary Figures S7A–D**).

These data demonstrate that prolonged treatment with CXCR4 antagonist X4-185 corrected splenic B-cell abnormalities in *Cxcr4^+/1013^* mice, while normalizing B-cell lymphopenia in the peripheral blood.

## 4 Discussion

In the present study, we have investigated the impact of chronic dosing of the orally bioavailable CXCR4 antagonist X4-185 on leukocyte trafficking and distribution in primary and secondary lymphoid organs in a mouse model of WHIM syndrome harboring the heterozygous *Cxcr4^1013^* mutation, which is frequently observed in patients with WHIM syndrome. We provide evidence that CXCR4 antagonism corrected peripheral blood leukopenia and mobilized functional neutrophils without affecting BM granulopoiesis in *Cxcr4^+/1013^* mice. In addition, CXCR4 antagonism restored splenic T and B cell abnormalities in *Cxcr4^+/1013^* mice. These findings provide a more comprehensive understanding of how targeting the dysregulated CXCR4 signaling pathway can mitigate the pathogenesis of WHIM syndrome.

WHIM syndrome is predominantly caused by GOF mutations in the gene coding the CXCR4 receptor that leads to severe peripheral leukopenia (2, 3, 29). A recent phase 3 trial has shown that blockade of CXCR4 with mavorixafor, an orally bioavailable and selective CXCR4 antagonist, demonstrated clinically meaningful increases in absolute neutrophil and lymphocyte counts in patients with WHIM syndrome, leading to the recent FDA approval of XOLREMDI^®^ (mavorixafor). Moreover, treatment with mavorixafor was shown to reduce the annualized infection rate in patients with WHIM syndrome (24). In agreement with these observations in patients with WHIM syndrome, we found that CXCR4 antagonism efficiently corrected the quantitative defects of circulating leukocyte counts in *Cxcr4^+/1013^* mice. It is worth noting that CXCR4 antagonism had a more pronounced effect on leukocyte mobilization in *Cxcr4^+/1013^* than in WT mice (**Table 1**). This observation raises several hypotheses that are not mutually exclusive: (1) *Cxcr4^+/1013^* mice may have a greater leukocyte reservoir than WT mice due to sequestration of leukocytes in different tissues; (2) CXCR4 antagonism may mobilize cells from different and/or multiple tissue compartments in *Cxcr4^+/1013^* mice compared with WT mice. Additional studies are required to fully address these questions.

In patients with WHIM syndrome, CXCR4 GOF mutations cause increased retention of neutrophils in the BM, and this process is thought to contribute to circulating neutropenia (2, 10). *Cxcr4^+/1013^* mice exhibited moderate neutropenia; however, this happened in the context of normal neutrophil maturation and was associated with neither accumulation of neutrophils nor increased apoptosis in the BM (15). This observation suggests that in *Cxcr4^+/1013^* mice, neutrophils may preferentially accumulate in other tissues, rather than in BM, or that the clearance of neutrophils by tissue-resident macrophages could be differentially regulated in mice versus humans in this WHIM mouse model. Nonetheless, we observed that chronic treatment with a CXCR4 antagonist led to an increased mature neutrophil count in the blood, which correlated with a decreased mature neutrophil count in the BM of *Cxcr4^+/1013^* and WT mice. Our data implicate the BM as a critical source of CXCR4 antagonist-mobilized neutrophils, which is consistent with previous reports (27, 28, 30). Whether CXCR4 antagonism also leads to a redistribution of neutrophils from different sources other than BM warrants further investigations. Notably, we also observed that continuous dosing with a CXCR4 antagonist did not appear to hinder the differentiation and maturation of neutrophils within the BM of both *Cxcr4^+/1013^* and WT mice, thereby effectively facilitating the mobilization of neutrophils into peripheral blood without disrupting their production and maturation in BM.

Increased risk of infection in patients with WHIM syndrome is thought to be associated with the reduced levels of circulating neutrophils and/or defects in neutrophil function. Surprisingly, little is known about neutrophil function in the context of WHIM syndrome. Here, we found that blood *Cxcr4^+/1013^* neutrophils were able to produce similar ROS levels and to phagocytose *S. aureus* BioParticles as efficiently as their WT counterparts, thus suggesting that *Cxcr4^+/1013^* neutrophil function remained intact. Congruent with this, neutrophil function in most WHIM patients with reported functional studies showed normal levels of phagocytosis, bacterial killing activity, and ROS production in response to PMA or bacterial-derived polysaccharide complexes (3, 23, 31). Moreover, we showed that CXCR4 antagonist treatment increased neutrophil counts in blood, while preserving their effector functions. Our findings suggest that the susceptibility of patients with WHIM syndrome to infection may be more a consequence of neutropenia rather than a defect in neutrophil function. Our data provide additional evidence to support the notion that CXCR4 antagonist treatment effectively mobilizes more functional neutrophils, that may, in turn, help patients with WHIM syndrome to overcome the recurrent infections commonly observed.

It was recently reported that CXCR4 GOF mutations lead to a more severe depression of circulating CD8^+^ T-cell counts compared with CD4^+^ T cells in patients with WHIM syndrome and in a WHIM mouse model (18). In the phase 3 trial in patients with WHIM syndrome, treatment with CXCR4 antagonist mavorixafor increased and corrected peripheral blood CD4^+^ and CD8^+^ T-cell lymphopenia (32, 33). The current data presented here with *Cxcr4^+/1013^* mice corroborated these previous observations in patients with WHIM syndrome. Specifically, we found that *Cxcr4^+/1013^* mice exhibited reduced T-cell counts in peripheral blood and spleen compared with WT mice, with CD8^+^ T cells being more affected. CXCR4 antagonism reversed peripheral T-cell lymphopenia and restored the CD4/CD8 T-cell ratio in *Cxcr4^+/1013^* mice. Importantly, we uncovered that chronic treatment with a CXCR4 antagonist was able to normalize splenic CD8^+^ T-cell counts and splenic CD4/CD8 T-cell ratio in *Cxcr4^+/1013^* mice. T-cell lymphopenia in *Cxcr4^+/1013^* and *Cxcr4^+/1000^* mice was attributed to sequestration of T cells in primary lymphoid organs, or defects in T-cell trafficking between bloodstream and secondary lymphoid organs (15, 17, 18). Correction of splenic T-cell counts and distribution abnormalities by CXCR4 antagonism may be facilitated by the release of sequestered T cells from primary lymphoid organs and renewed migration to peripheral lymphoid organs. However, we cannot exclude that CXCR4 antagonist treatment may have a direct impact on the survival of splenic T cells in *Cxcr4^+/1013^* mice. Further work will be required to address this point.

In addition to correcting T-cell lymphopenia, we also observed that CXCR4 antagonist treatment normalized peripheral blood B-cell counts in *Cxcr4^+/1013^* mice. Moreover, we uncovered that chronic treatment with a CXCR4 antagonist X4-185 for four weeks corrected splenic B-cell alterations in *Cxcr4^+/1013^* mice. In line with our data, chronic treatment with the CXCR4 antagonist X4-185 for three weeks was reported to increase and normalize B-cell numbers in blood and spleen in an independent WHIM mouse model harboring the *Cxcr4^1000^* mutation (17). Our findings are thus consistent with the recent phase 3 trial results in patients, in which mavorixafor treatment resulted in correction of circulating B-cell lymphopenia (32, 33). Interestingly, we further showed that CXCR4 antagonist X4-185 normalized the frequencies and numbers of splenic MZ and FO B cells in *Cxcr4^+/1013^* mice. The precise mechanisms by which CXCR4 antagonism corrected the splenic B-cell abnormalities in this WHIM mouse model remain unclear. However, it is possible that the CXCR4 antagonist may act directly or indirectly on the splenic stromal cell compartment, favoring the proper trafficking, compartmentalization, and/or maturation of B cells within the spleen. CXCR4 antagonism may also normalize B-cell development and subsequently correct peripheral B-cell count in *Cxcr4^+/1013^* mice, as was recently shown in the *Cxcr4^+/1000^* model (17). Interestingly, we observed that chronic treatment with a CXCR4 antagonist does not alter antibody production in WT and *Cxcr4^+/1013^* mice, suggesting that while the distribution of cells from the B lineage is corrected, their functionality remains unchanged. The ability of CXCR4 antagonists to normalize the abnormalities observed in the splenic B-cell populations may be compound specific and related to the unique tissue distribution properties and residence time of each compound bound to the CXCR4 receptor, as well as the duration of treatment of the antagonist. Supporting these hypotheses, we found that chronic treatment with plerixafor (AMD3100) was unable to correct splenic FO and MZ B-cell defects observed in *Cxcr4^+/1013^* mice. This is in contrast to the effects observed with the orally bioavailable CXCR4 antagonist X4-185.

In summary, this study provides more comprehensive evidence that CXCR4 antagonism can effectively correct WHIM-associated neutrophil and lymphocyte abnormalities in primary and secondary lymphoid organs in a mouse model of WHIM syndrome. Specifically, CXCR4 antagonism normalized peripheral blood leukopenia and restored abnormal splenic B- and T-cell counts and distribution in *Cxcr4^+/1013^* mice. CXCR4 antagonism mobilized functional neutrophils from BM without disrupting normal granulopoiesis. These findings significantly advance our understanding of how targeting the dysregulated CXCR4 signaling pathway can ameliorate the leukocyte pathogenesis of WHIM syndrome.

## Data Availability

The original contributions presented in the study are included in the article/**Supplementary Material**. Further inquiries can be directed to the corresponding authors.
