# Supplementary material for: CXCR4 antagonism ameliorates leukocyte abnormalities in a preclinical model of WHIM syndrome
Source: Front Immunol. 2024 Nov 11;15:1468823. doi: 10.3389/fimmu.2024.1468823 (PMC11586337; doi:10.3389/fimmu.2024.1468823)
Supplement: Supplementary file 1 [file DataSheet1.docx]

Supplementary Material

[1 Supplementary Methods 2](#_Toc171327214)

[2 Supplementary Tables 3](#_Toc171327215)

[3 Supplementary Figure Legends 5](#_Toc171327216)

[4 Supplementary Figures 7](#_Toc171327217)

# 1 Supplementary Methods

## 1.1 Ligand-binding inhibition assay

Jurkat cells were washed once with assay buffer (Hanks’ balanced salt solution + 20 mM 4-(2-hydroxyethyl)-1-piperazineethanesulfonic acid buffer + 0.2% bovine serum albumin, pH 7.4) and then incubated for 15 minutes at room temperature (RT) with test compound diluted in assay buffer at dose-dependent concentrations. Subsequently, human C-X-C motif chemokine ligand 12–AlexaFluor647 (CXCL12-AF647; 26 ng/mL) (Almac, Craigavon, Northern Ireland, UK) was added to the compound-preincubated cells. The cells were incubated for 30 minutes at RT. Thereafter, the cells were washed twice in assay buffer, fixed with 1% paraformaldehyde in phosphate-buffered saline, and analyzed by flow cytometry (CytoFLEX Flow Cytometer, Beckman Coulter Life Sciences, High Wycombe, Buckinghamshire, UK). Mean fluorescence intensity of CXCL12-AF647 was determined (FCS Express™ software, De Novo Software, Pasadena, CA, USA). The percentage of inhibition was calculated according to the formula:

$$\left( 1-\frac{MFI-{MFI}_{NC}}{{MFI}_{PC}-{MFI}_{NC}} \right)\times100$$

where MFI is the mean fluorescence intensity of cells in the presence of an inhibitor, MFI_NC_ is mean fluorescence intensity of cells in the absence of the ligand, and MFI_PC_ is the mean fluorescence intensity of cells in the presence of the ligand alone.

For receptor occupancy experiments, Jurkat cells were preincubated with compounds at their 90% inhibition concentration for ligand-binding inhibition for 15 minutes at RT. Then, cells were washed once in assay buffer, and the compounds were let to dissociate at RT for the various predefined timepoints. Subsequently, human CXCL12-AF647 was added to the preincubated cells. After that, cells were washed twice with assay buffer and fixed with 1% paraformaldehyde. MFI of CXCL12-AF647 was determined (FCS Express™). The staining intensity was inversely proportional to the fraction of receptors occupied by the compound at the respective timepoints. Receptor occupancy half-life was calculated using the “Dissociation - One phase exponential decay” method (GraphPad Prism 10 software, GraphPad Software, Boston, MA, USA).

## 1.2 Pharmacokinetic studies

Male C57BL/6J mice (JH Laboratory Animal Co. LTD), weighing 23–25 g, were quarantined for 1 week before the experiment. Mice had free access to food and water. X4-185 dissolved in 50 mM citrate buffer (pH 4.0) was given to mice via oral gavage (N = 6). The animals were anesthetized via isoflurane at the designated timepoints (before dosing and 4, 8, and 24 hours after dosing). Approximately 30 µL of blood was taken from the animals via facial vein into K2EDTA tubes. Blood samples were put on wet ice and centrifuged to obtain plasma samples (2000 g, 5 minutes, under 4 ℃) within 15 minutes. Plasma samples were stored at approximately −70 ℃ until analysis. The plasma samples were analyzed by liquid chromatography–tandem mass spectrometry (LC-MS/MS-28) (Triple Quad™ 6500^+^ System, SCIEX, Framingham, MA, USA). An aliquot of 5 µL sample was added to 500 µL acetonitrile, which contained 50 ng/mL of an insulin secretagogue (glipizide). The mixture was vortexed for 10 minutes and centrifuged at 5800 rpm for 10 minutes. An aliquot of 0.5 µL supernatant was injected for LC-MS/MS analysis. Pharmacokinetic parameters were estimated via a noncompartmental model using Phoenix WinNonlin™ 6.4 (Certara, USA).

# 2 Supplementary Tables

## SUPPLEMENTARY TABLE S1 Antibodies used for flow cytometry.

| Mouse flow cytometry reagent | | | |
| --- | --- | --- | --- |
| Reagent | | | Supplier |
| BD Horizon™ Streptavidin | | | BD Bioscience |
| eBioscience™ Fixable Viability Dye eFluor™ | | | Thermofisher |
| Mouse flow cytometry antibody | | | |
| Antibody | Clone | Host/isotype | Supplier |
| Anti-CD16/32  (Fc Block^TM^) | 2.4G2 | Rat IgG2b, κ | BD Bioscience |
| Anti-CD11b | M1/70 | Rat IgG2b, κ | Thermofisher |
| Anti-CD19 | 1D3 | Rat IgG2a, κ | BD Bioscience |
| Anti-CD21 | 7G6 | Rat IgG2b, κ | BD Bioscience |
| Anti-CD23 | B3B4 | Rat IgG2a, κ | BioLegend |
| Anti-CD3 | 17A2 | Rat IgG2b, κ | BD Bioscience |
| Anti-CD4 | RM4-5 | Rat IgG2a, κ | BD Bioscience |
| Anti-CD45R/B220 | RA3-6B2 | Rat IgG2a, κ | BD Bioscience |
| Anti-CD8a | 53-6.7 | Rat IgG2a, κ | BD Bioscience |
| Anti-Ly-6C | AL-21 | Rat IgM, κ | BD Bioscience |
| Anti-Ly-6G-C (Gr-1) | RB6-8C5 | Rat IgG2b, κ | BD Bioscience |
| Anti-CXCR4 | 2B11 | Rat IgG2b, κ | BD Bioscience |

## SUPPLEMENTARY TABLE S2 Pharmacokinetic parameters following single oral administration of X4-185 and subcutaneous injection of AMD3100 in WT mice.

| Parameter | X4-185 | AMD3100* |
| --- | --- | --- |
| Dose (mg/kg) | 10 | 5 |
| T_max_ (h) | 4 | 0.25 |
| C_max_ (ng/mL) | 55.8 | 10951 |
| T_1/2_ (h) | 6.03 | 0.75 |
| AUC_last_ (hr×ng/mL) | 503 | 8823 |
| Vz (mL/kg) | 17200 | 612 |

***** Pharmacokinetic data of AMD3100 were curated from European Medicines Agency. CHMP ASSESSMENT REPORT FOR Mozobil (2009). [https://www.ema.europa.eu/en/documents/
assessment-report/mozobil-epar-public-assessment-report_en.pdf](https://www.ema.europa.eu/en/documents/assessment-report/mozobil-epar-public-assessment-report_en.pdf) [Accessed 10 July 2024]. AUC_last_, the total area under the curve from time zero to the last evaluated time point; C_max_, maximum concentration of drug in blood plasma; T_1/2_, half-life; T_max_, time after drug administration at which peak plasma concentration occurs; Vz, volume of distribution.

# 3 Supplementary Figure Legends

## SUPPLEMENTARY FIGURE S1

Membrane CXCR4 expression in peripheral blood and bone marrow neutrophils, B-cells, CD4^+^-T and CD8^+^-T cells in *Cxcr4^+/1013^* and *Cxcr4^WT^* mice. CXCR4 expression was assessed by flow cytometry. The geometrical mean of fluorescence is indicated. Data (mean + SEM) were from two independent experiments with 5-7 mice per group. Statistics were calculated using the nonparametric Mann-Whitney test. CXCR4, C-X-C chemokine receptor 4; SEM, standard error of the mean; WT, wild-type.

## SUPPLEMENTARY FIGURE S2

Effect of CXCR4 antagonist X4-185 on RBC and platelet counts in *Cxcr4^+/1013^* and *Cxcr4^WT^* mice. Absolute (**A**) RBC and (**B**) platelet counts were determined in the blood of *Cxcr4^WT^* and *Cxcr4^+/1013^* mice 3 hours after last dose on day 7. Data (mean + SEM) were from two independent experiments with 6–7 mice per group. CXCR4, C-X-C chemokine receptor 4; RBC, red blood cell; SEM, standard error of the mean; Veh., vehicle; WT, wild-type.

## SUPPLEMENTARY FIGURE S3

Effect of CXCR4 antagonism on splenic T-cell compartment in *Cxcr4^+/1013^* mice. Absolute (**A**) CD4^+^ T-cell counts, (**B**) CD8^+^ T-cell counts, and (**C**) CD4/CD8 T-cell ratio were determined in the blood 3 hours after the last dose on day 7. Total (**D**) CD4 T-cell numbers, (**E**) CD8 T-cell numbers, and (**F**) CD4/CD8 T-cell ratio were determined in the spleen of WT and *Cxcr4^+/1013^* mice after 7 days of X4-185 treatment. Data (mean + SEM) were from two independent experiments with 6–10 mice per group. Statistics were calculated using the nonparametric Mann-Whitney test, two-sided. * p < 0.05, ** p < 0.01, *** p < 0.001. CXCR4, C-X-C chemokine receptor 4; SEM, standard error of the mean; Veh., vehicle; WT, wild-type.

## SUPPLEMENTARY FIGURE S4

Effect of CXCR4 antagonist X4-185 on immunoglobulin (IgM and IgG1) levels in *Cxcr4^+/1013^* and *Cxcr4^WT^* mice. (**A**) IgM and (**B**) IgG1 levels were determined in the serum of *Cxcr4^WT^* and *Cxcr4^+/1013^* mice 3 hours after last dose on day 7 and day 28. Data (mean + SEM) were from two independent experiments with 6–12 mice per group. Statistics were calculated using the nonparametric Mann-Whitney test, two-sided. * p < 0.05. CXCR4, C-X-C chemokine receptor 4; SEM, standard error of the mean; Veh., vehicle; WT, wild-type.

## SUPPLEMENTARY FIGURE S5

Impact of CXCR4 antagonist X4-185 on (**A**) spleen weight and (**B**) total splenocytes in *Cxcr4^+/1013^* and *Cxcr4^WT^* mice after 28 days of X4-185 treatment. Data (mean + SEM) were from two independent experiments with 6–10 mice per group. Statistics were calculated using the nonparametric Mann-Whitney test, two-sided. * p < 0.05. CXCR4, C-X-C chemokine receptor 4; SEM, standard error of the mean; Veh., vehicle; WT, wild-type.

## SUPPLEMENTARY FIGURE S6

Receptor occupancy half-life of CXCR4 antagonist. Jurkat cells were preincubated with CXCR4 antagonists at their 90% inhibition concentration, determined from ligand-binding inhibition assays (X4-185, 10 nM; AMD3100, 2 nM). Cells were left to dissociate for the various predefined timepoints before human CXCL12-AF647 was added to preincubated cells. The MFI of CXCL12-AF647 was determined, and the receptor occupancy half-life was calculated. The experiment with AMD3100 was performed in presence of zinc (<https://go.drugbank.com/salts/DBSALT002325>). Data (mean + SEM) were from two independent experiments. CXCL12-AF647, human C-X-C motif chemokine ligand 12–AlexaFluor647; CXCR4, C-X-C chemokine receptor 4; MFI, mean fluorescence intensity; SEM, standard error of the mean.

## SUPPLEMENTARY FIGURE S7

Impact of CXCR4 antagonist AMD3100 on splenic B cell in *Cxcr4^WT^* and *Cxcr4^+/1013^* mice. The frequency of (**A**) FO or (**B**) MZ cells and total numbers of (**C**) FO or (**D**) MZ B cells were determined in the spleen of *Cxcr4^+/1013^* and *Cxcr4^WT^* mice after 21 days of AMD3100 treatment. Data (mean + SEM) were from two independent experiments with 8-13 mice per group. Statistics were calculated using the nonparametric Mann-Whitney test, two-sided. * p < 0.05, ** p < 0.01, *** p < 0.001. CXCR4, C-X-C chemokine receptor 4; FO, follicular; MZ, marginal zone; SEM, standard error of the mean; Veh., vehicle; WT, wild-type.

# 4 Supplementary Figures


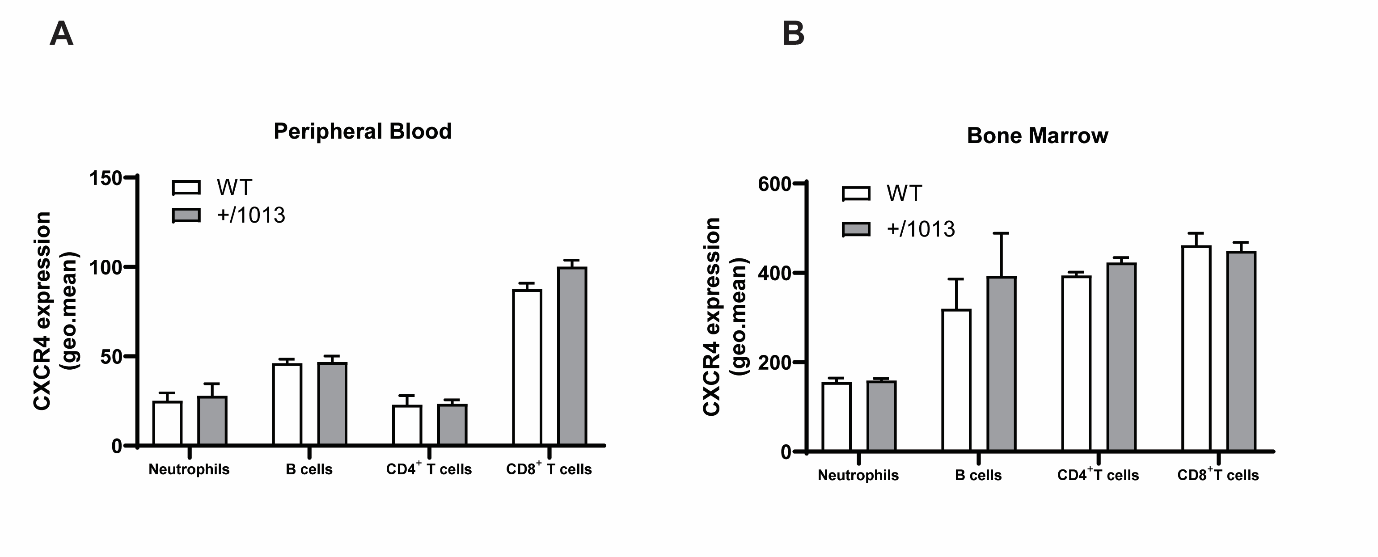


**SUPPLEMENTARY FIGURE S1**


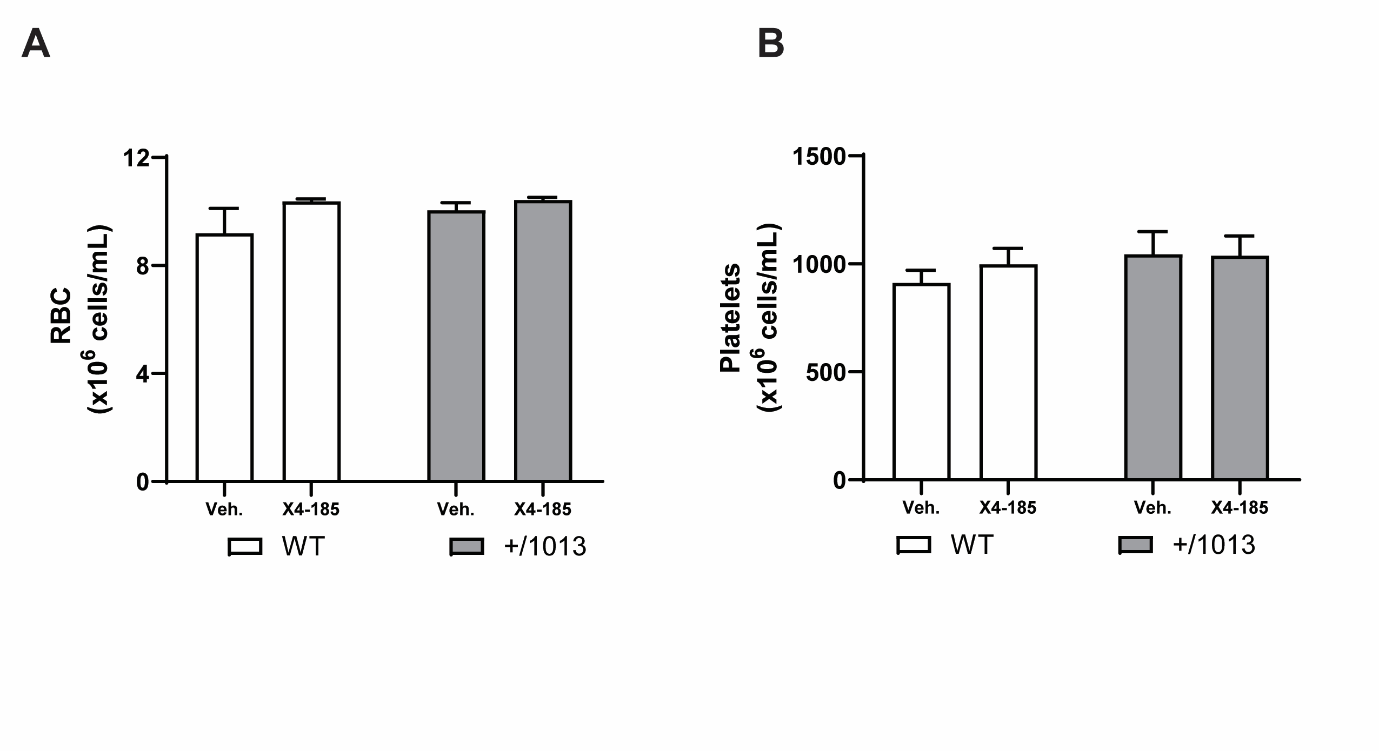


**SUPPLEMENTARY FIGURE S2**


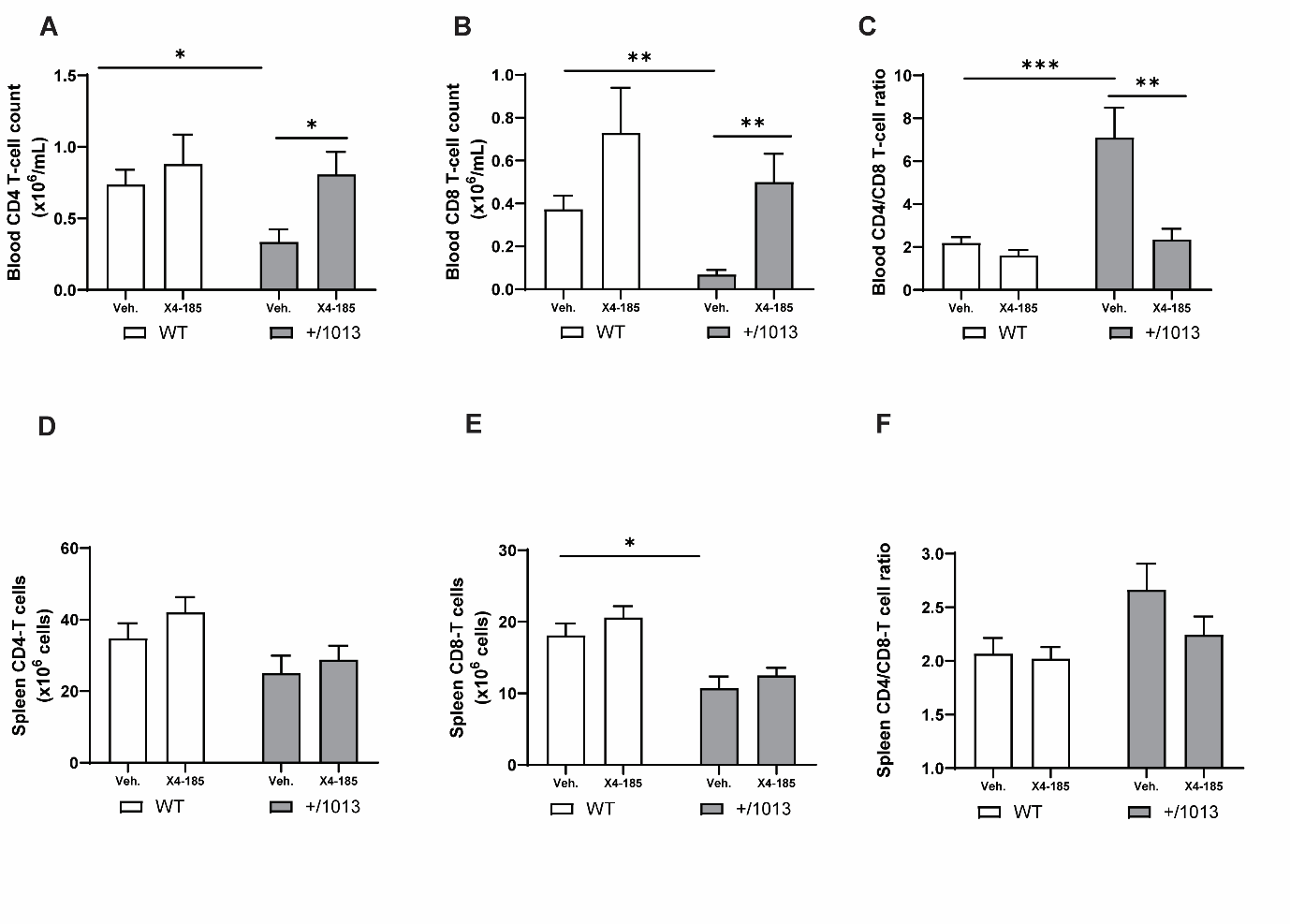
**SUPPLEMENTARY FIGURE S3**


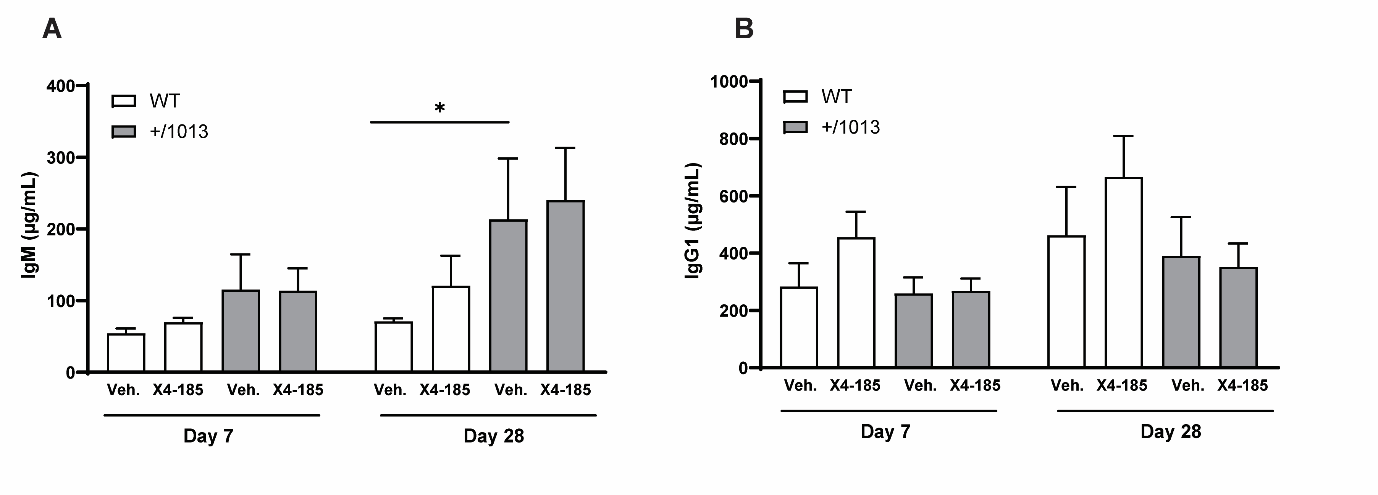
**SUPPLEMENTARY FIGURE S4**


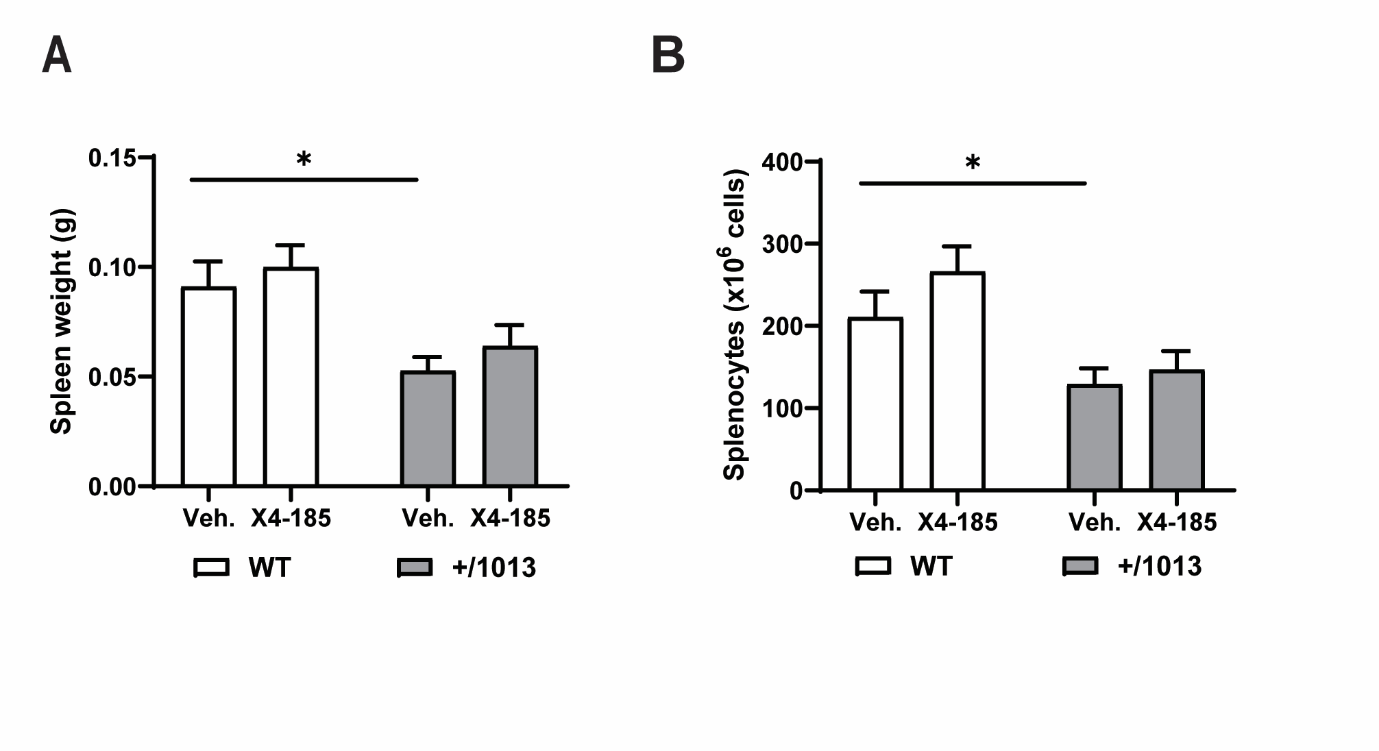


**SUPPLEMENTARY FIGURE S5**


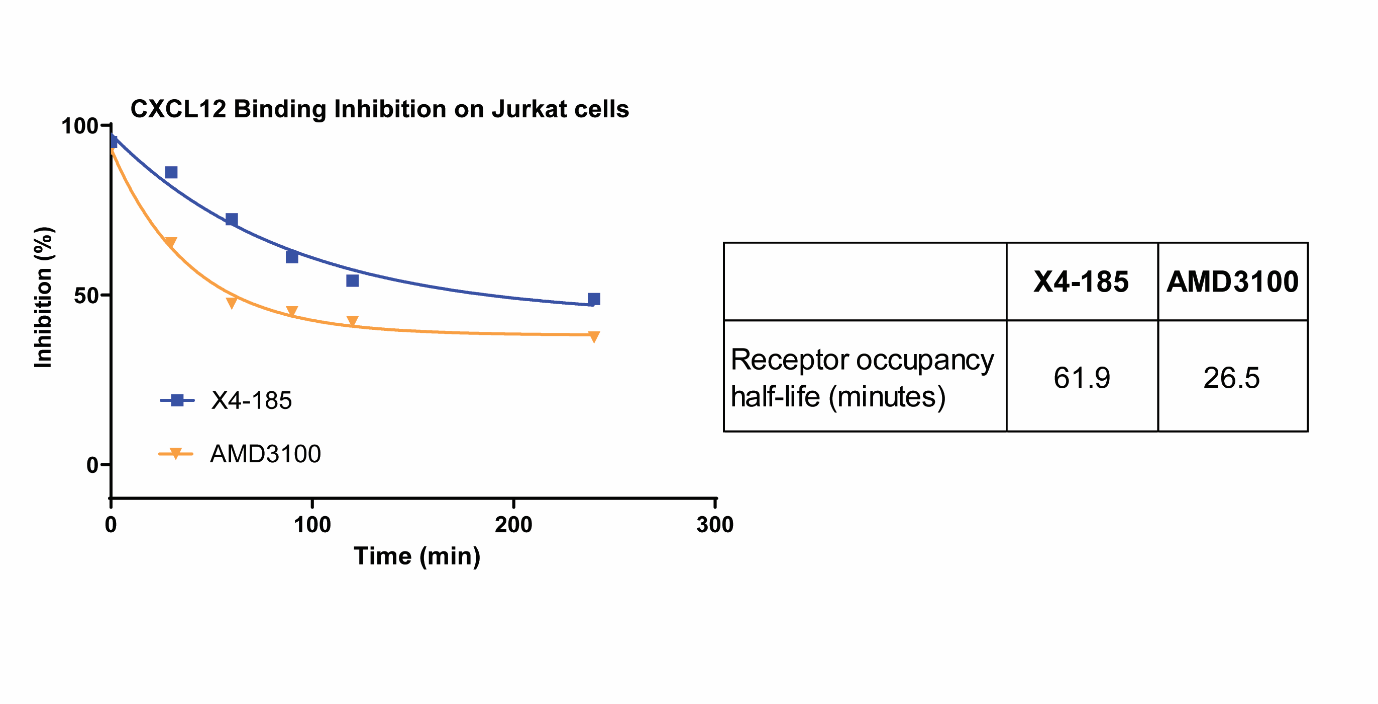


**SUPPLEMENTARY FIGURE S6**


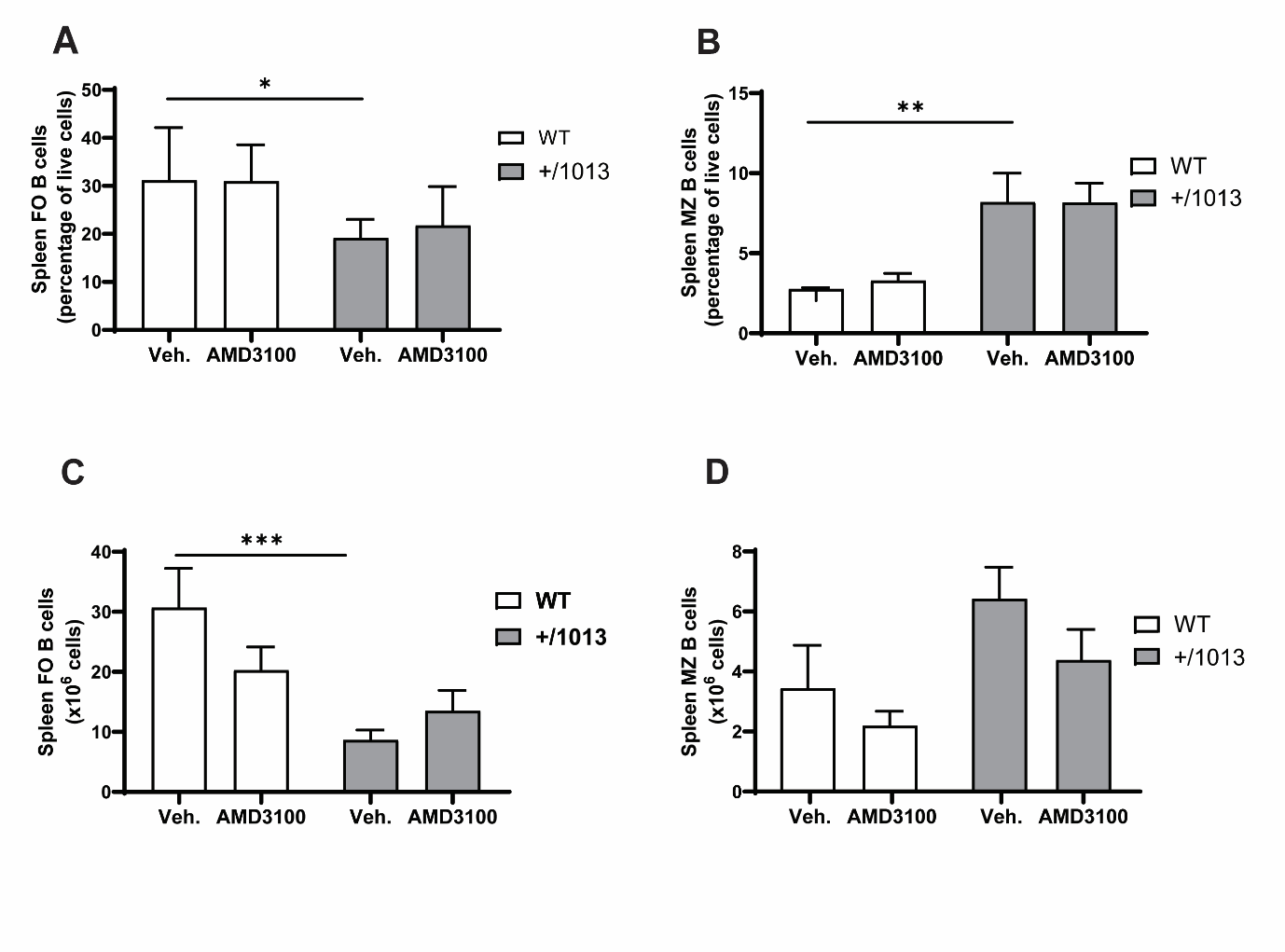
**SUPPLEMENTARY FIGURE S7**
